# Pediatric Noninfectious Uveitis in a Tertiary Referral Center in Jordan: Clinical Spectrum and Immunomodulatory Treatment

**DOI:** 10.7759/cureus.25841

**Published:** 2022-06-11

**Authors:** Raed Alzyoud, Motasem Alsuwaiti, Hiba Maittah, Boshra Aladaileh, Mohammed Nobani, Ayman Farhan, Hadeel Alqurieny, Ahmed Khatatbeh, Zeyad Habahbeh

**Affiliations:** 1 Allergy, Immunology, and Rheumatology, Queen Rania Children’s Hospital, Amman, JOR; 2 Dentistry, Queen Rania Children’s Hospital, Amman, JOR; 3 Endocrinology, Queen Rania Children’s Hospital, Amman, JOR; 4 Ophthalmology, King Hussein Medical Center, Amman, JOR

**Keywords:** noninfectious uveitis, immunomodulatory, jordan, idiopathic uveitis, juvenile idiopathic arthritis

## Abstract

Objectives

This study aims to describe the clinical, etiological, and treatment features of noninfectious uveitis in Jordanian children in a single center.

Methods

A retrospective, observational analysis of medical records of pediatric patients who were diagnosed with noninfectious uveitis from 2015 to 2020 at pediatric rheumatology and ophthalmology clinics at Queen Rania Children’s Hospital, Amman, Jordan, was conducted. All patients were below 14 years of age at diagnosis. The collected data included age at diagnosis, anatomical location of uveitis, laterality, associated systemic disease, and used medications.

Results

Overall, 96 patients were included in this cohort (41 males and 55 females), with a mean age at diagnosis of 8.4±2.4 years. Anterior uveitis (44.8%) was the commonest anatomical location. Based on laterality, bilateral uveitis was reported in 59.3% of all patients. Idiopathic uveitis (46.9%) and juvenile idiopathic arthritis-associated uveitis (JIAU) (35.5%) were the most common diagnoses. Of the children with idiopathic uveitis, 47% had panuveitis, while 61.7% of the children with JIAU had chronic anterior uveitis. Posterior synechiae were the most common complication (12.5%). Patients with refractory uveitis received infliximab (29.1%) and adalimumab (4.1%).

Conclusion

To the best of our knowledge, this is the first report on noninfectious uveitis in Jordanian children. Compared with other regional and international published reports, JIAU and idiopathic uveitis were the most common diagnoses. To obtain more details on noninfectious uveitis characteristics, a population-based rather than a single-center study is needed in Jordan.

## Introduction

Uveitis involves inflammation of the uveal tract. Rheumatological disorders in childhood are relatively common, but children with uveitis account for only 5%-10% of all uveitis patients [1]. Despite the lower incidence of pediatric uveitis compared to adults, there are many considerations in childhood-onset uveitis, such as few presenting symptoms, an insidious course, difficulties in eye examination, and a higher risk of vision-threatening complications. These factors make the diagnosis and treatment more challenging [2].

Uveitis has been classified in different ways. Anatomical classifications divide the condition into anterior, intermediate, posterior, and panuveitis. In contrast, onset-based classifications divide it into acute, recurrent, and chronic, and etiology-based classifications divide it into those due to infectious and those due to noninfectious causes. There is a wide range of noninfectious causes of pediatric uveitis, such as juvenile idiopathic arthritis (JIA), Behçet’s disease (BD), sarcoidosis, vasculitis, and masquerading syndromes, but idiopathic uveitis is still the most common cause [3].

The management strategy for noninfectious uveitis in children needs multidisciplinary diagnostic and therapeutic approaches involving pediatric rheumatologists and expert ophthalmologists. Treatment options are guided by the severity and anatomical involvement of the disease with a stepladder plan using topical or systemic steroids, classic or biological immunomodulatory agents, or a combination of all [4]. This study aims to evaluate pediatric noninfectious uveitis and report clinical manifestations, uveitis patterns, therapeutic options, and outcomes in a single center in Jordan.

## Materials and methods

Patients and materials

This study involved a retrospective review of medical records of pediatric patients who were diagnosed with chronic noninfectious uveitis in pediatric rheumatology and ophthalmology clinics at Queen Rania Children’s Hospital, the only tertiary children’s hospital in Jordan between 2015 and 2020. The inclusion criteria were children 14 years old or less at the time of diagnosis based on the Jordanian Ministry of Health age limit of pediatrics, confirmed diagnosis of chronic noninfectious uveitis, and inflammation that persisted for more than six weeks. The exclusion criteria were acute uveitis, inflammation that persisted for less than six weeks, patients who had a diagnosis other than infectious uveitis or masquerade syndromes, patients who were lost follow-up before remission, and patients with incomplete data. Active inflammation and inactive disease were defined using the Standardization of Uveitis Nomenclature (SUN) guidelines [5]. The study was approved by the Royal Medical Services Human Research Ethics Committee (approval number 12/2021).

Data collection and statistics

**Figure 1:**
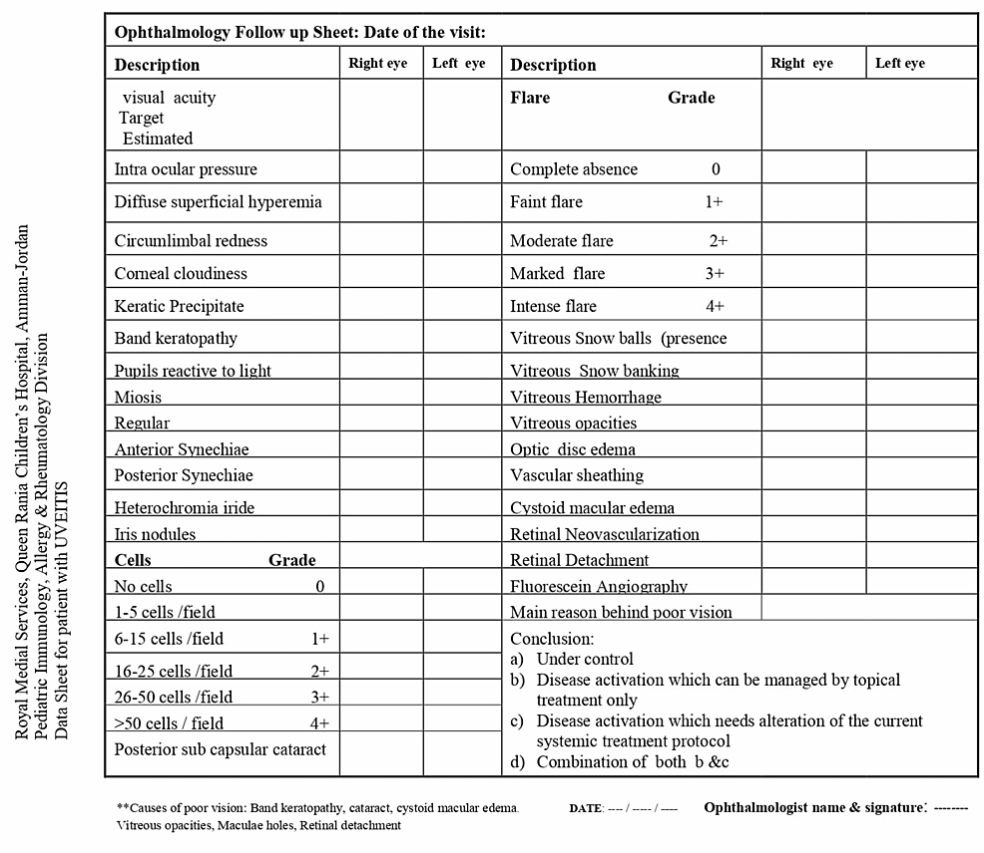
Data sheet for patients with uveitis

A dedicated intake sheet was designed for every patient and filled in at diagnosis and subsequent visits. The data sheet included patient demographics, diagnostic workup, presenting symptoms, detailed physical examination including a thorough musculoskeletal examination, anatomical location, detailed slit-lamp examination to assess severity, laterality, associated systemic disease, treatment plan, and complications (Figure 1). All data sheets were maintained in paper files for each patient in Pediatric Immunology and Rheumatology Unit at Queen Rania Children’s Hospital. Patients with noninfectious uveitis were classified based on anatomical involvement into anterior, intermediate, posterior, or panuveitis, laterality into unilateral or bilateral, and severity into mild, moderate, or severe. Diagnostic workup at first presentation included complete blood count (CBC), liver function test (LFT), urea and creatinine, electrolytes, C-reactive protein (CRP), erythrocyte sedimentation rate (ESR), antistreptolysin O titer (ASOT), viral screen by polymerase chain reaction PCR in blood for cytomegalovirus (CMV), Epstein-Barr virus (EBV), herpes simplex virus (HSV), human leukocyte antigen (HLA) B27, B5, angiotensin-converting enzyme (ACE) level, urine analysis, chest X-ray, sacroiliac joint X-ray, magnetic resonance image (MRI) when indicated, purified protein derivative (PPD) test, pathergy test in cases of recurrent oral aphthae, antinuclear antibody (ANA), and rheumatoid factor (RF). Statistical analysis was done using the Statistical Package for the Social Sciences (SPSS) version 18.0 (IBM Corp., Armonk, NY, USA).

## Results

A total of 96 patients with noninfectious uveitis below 14 years were included in our cohort. The patients comprised 41 (42.7%) males and 55 (57.3%) females, and the mean age at the time of diagnosis was 8.4±2.4 years. Table 1 demonstrates the patients’ demographics, age at diagnosis, anatomical classification, frequency, laterality, common presenting symptoms, disease severity, and common etiological causes. The most common anatomical location was anterior uveitis, which was diagnosed in 43 (44.8%) patients, and juvenile idiopathic arthritis (JIA) caused half of these (21/43, 48.8%). Panuveitis was the second most common anatomical classification in 40 patients (41.7%), followed by intermediate and posterior uveitis in eight (8.3%) and five (5.2%) patients, respectively. Bilateral uveitis was observed in 57 (59.3%) patients, and unilateral uveitis was observed in 39 (40.6%) patients.

**Table 1:** Demographics of 96 patients based on the anatomical location of intraocular inflammation JIA: juvenile idiopathic arthritis, VKH: Vogt-Koyanagi-Harada, JPsA: juvenile psoriatic arthritis

		Anterior (n=43 (44.8%))	Intermediate (n=8 (8.3%))	Posterior (n=5 (5.2%))	Panuveitis (n=40 (41.7%))	Total (N=96 (100%)) (n (%))
Gender	Male	15	3	1	22	41 (42.7)
	Female	28	5	4	18	55 (57.3)
Mean age	(Years)	9.14	8	8.3	8.5	8.4±2.4
Laterality	Unilateral	16	3	4	16	39 (40.6)
	Bilateral	27	5	1	24	57 (59.3)
Presenting symptoms	Photophobia	5	0	2	3	10 (10.4)
	Red eye	13	3	0	8	24 (25)
	Blurred vision	12	3	4	21	40 (41.6)
	None	11	0	0	6	17 (17.7)
Disease severity at diagnosis	Mild	9	0	1	1	11 (11.5)
	Moderate	25	5	2	5	37 (38.5)
	Severe	9	3	3	33	48 (50)
Etiology	Idiopathic	18	7	5	15	45 (46.9)
	JIA	21	0	0	13	34 (35.5)
	Behçet’s disease	3	1	0	7	11 (11.4)
	VKH disease	0	0	0	3	3 (3.1)
	JPsA	1	0	0	2	3 (3.1)

The most frequent presenting feature was blurred vision (41.6%), followed by eye redness (25%), photophobia (10.4%), and strabismus (6.2%). Seventeen patients (17.7%) were asymptomatic and diagnosed during routine screening for uveitis. The uveitis severity was classified as mild, moderate, and severe uveitis at the time of diagnosis. Half of the patients were classified as severe at the diagnosis, and panuveitis caused 68.8% of the severe cases. Moderate severity was seen in 37 (38.5%) patients, and two-thirds (25 patients, 67.5%) had anterior uveitis. Mild severity was seen in only 11 (11.5%) patients.

The etiological causes of uveitis in our cohort were JIA-associated uveitis in 34 (35.5%) patients, followed by BD in 11 (11.4%) patients. Other underlying systemic diseases were Vogt-Koyanagi-Harada (VKH) disease in three (3.1%) patients, juvenile psoriatic arthritis (JPsA) in three (3.1%) patients, and unidentified underlying causes in 45 (46.9%) patients, who were labeled as idiopathic.

Table 2 shows treatment options that had been used on these patients. Topical treatments (steroids, mydriatics, and sub-tenon steroid injections) were used on 90 (93%) patients either alone or with systemic therapy. Systemic corticosteroids were used on all patients (either intravenous steroid pulses in 15 (15.6%) patients or tapered oral steroids in 81 (84.3%) patients). Among conventional disease-modifying antirheumatic drugs (DMARDs), methotrexate (MTX) was used on 67 (69.7%) patients, cyclosporine was used on three (3.1%) patients, and azathioprine was used on six (6.2%) patients. Among biological DMARDs, infliximab was used on 28 (29.1%) patients and adalimumab was used on four (4.1%) patients based on severity and responsiveness. Combination treatments of topical and systemic steroids, conventional DMARDs, and biological DMARDs were used on 67 (70%) patients. Surgical intervention was needed for only five patients (5.2%) for disease or treatment complications.

**Table 2:** Treatment options in 96 patients

	Anterior uveitis (n)	Intermediate uveitis (n)	Posterior uveitis (n)	Panuveitis (n)	Total (n (%))
Topical treatment	43	7	-	40	90 (93)
Oral systemic steroids	37	8	5	31	81 (84.3)
Intravenous steroid	2	3	1	9	15 (15.6)
Methotrexate	33	5	2	27	67 (69.7)
Cyclosporine	-	-	-	3	3 (3.12)
Azathioprine	-	3	-	3	6 (6.25)
Infliximab	11	3	-	14	28 (29.1)
Adalimumab	3	-	-	1	4 (4.1)

The best corrected visual acuity (BCVA) at presentation was classified according to severity as follows: less than 0.1 (4%), 0.1-0.3 (8%), 0.3-0.5 (16%), and 0.5-1.0 (72%). The mean BCVA was 0.6, while the mean BCVA at disease control or remission was 0.85. The ocular complications and outcomes of our cohort are listed in Table 3. During the follow-up period, 67 (69.7%) patients did not report any complications, while the most commonly reported complications were posterior synechiae in 12 (12.5%) patients, cataracts in six (6.2%) patients, and loss of vision in three (3.1%) patients.

**Table 3:** Ocular complications and outcomes in 96 patients IOP: intraocular pressure

Anatomical complication	Anterior (n=43)	Intermediate (n=8)	Posterior (n=5)	Panuveitis (n=40)	Total (n (%))
None	31	5	3	27	67 (69.1)
Cataract	4	2	0	0	6 (6.2)
Increased IOP	2	0	0	1	3 (3.1)
Retinal detachment	0	1	2	3	6 (6.2)
Posterior synechia	6	0	0	6	12 (12.3)
Visual loss	0	0	0	3	3 (3.1)

Among complications that needed surgical interventions, two patients had anterior uveitis complicated by band keratopathy, which needed keratectomy and ethylenediaminetetraacetic acid (EDTA) chelation. One patient had intermediate uveitis and a cataract and underwent vitrectomy, which resulted in improved vision and controlled intraocular inflammation. One patient with panuveitis had a retinal detachment and underwent scleral buckling surgery, while another with panuveitis had posterior synechiae and peripheral iridectomy.

## Discussion

Pediatric uveitis is still a challenging disease due to difficulties in early diagnosis, uncooperative subjects for an eye examination, the broad spectrum of infectious and noninfectious etiologies, treatment adherence and tolerance, late referral to specialists, and established complications due to untreated, persistent inflammation [6]. In this retrospective observational study, patients’ demographics, presenting symptoms, anatomical location of intraocular inflammation, laterality, underlying systemic diseases, complications, and treatment options were reviewed among patients diagnosed with noninfectious uveitis. Our study’s mean age at diagnosis was 8.4±2.4 years, which is similar to previously published reports [7,8]. Marino et al. [9] observed a significant female predominance (66%) among 118 pediatric patients (the median age was 7.4 years at diagnosis) with noninfectious uveitis. Conversely, our cohort showed no significant difference in gender (1:1.3), which is comparable to the report by Smith et al. [10], who reported on 285 pediatric patients with infectious and noninfectious uveitis (the median age at the time of diagnosis was 9.4 years). Furthermore, Thorne et al. [11] described the prevalence of noninfectious uveitis in pediatrics and adults and found no significant gender difference in 275 pediatric patients with noninfectious uveitis.

Noninfectious uveitis in children is often insidious in onset, and most of the children remain asymptomatic despite the severity of the inflammation. Less commonly, patients show symptoms such as episodes of pain, redness, photophobia, and/or blurred vision, which would prompt immediate eye examination [12]. The most common clinically presenting symptoms of uveitis in our cohort were blurred vision, red eye, and photophobia in 41.6%, 25%, and 10.4%, respectively, while 17.7% were asymptomatic and diagnosed during routine screening or as a workup of a disease presenting with extraocular manifestations. Yalçındağ et al. [13] reported that 40.7% of pediatric patients diagnosed with noninfectious uveitis were asymptomatic and diagnosed on routine surveillance. Blurred vision and redness were the most frequent complaints at 28.1% and 20.7%, respectively. The higher rate of asymptomatic patients compared to our data can be explained by the higher rate of uveitis associated with systemic diseases (50%).

BenEzra et al. [14] reported that 29% of children did not have ocular symptoms despite very poor visual acuity detected during routine testing in school. Common symptoms were tearing and photophobia (24.3%), red eye (15.9%), and a drop in vision (12%). However, Marino et al. [9] studied the symptomaticity of noninfectious uveitis in children and demonstrated that non-JIA diagnosis, older age, and antinuclear antibody (ANA) negativity were associated with symptomatic uveitis. Younger children and those with ANA positivity were less likely to have symptoms.

A comparison of our study and previous regional and international reports [13,15-18] regarding the type of uveitis, laterality, most common etiologies, and complications are listed in Table 4. A predominance of anterior uveitis (44.8%) was found in our cohort, similar to most reports [15,17,18]. Khairallah et al. reported the same frequency of anterior and intermediate uveitis at 31.2%, while Yalçındağ et al. reported slightly higher intermediate and anterior uveitis rates of 34.2% and 32.8%, respectively. Bilateral ocular involvement was found in 59.3% of our patients, comparable to data from Lebanon, Tunisia, and Italy [15,16,18]. Bilaterality accounts for most patients from Turkey and France at 86.6% and 79%, respectively [13,17].

**Table 4:** Comparison of the current study with regional and international data Pt: patients, JIAU: juvenile idiopathic arthritis-associated uveitis, NA: not applicable

		Current study	Al-Haddad (Lebanon) [15]	Khairallah (Tunisia) [16]	Yalçındağ (Turkey) [13]	Morelle (France) [17]	Paroli (Italy) [18]
	Number of Pt	96	49	64	76	147	257
Types of uveitis	Anterior	44.8%	40.8%	31.2%	32.8%	93%	47.8%
	Intermediate	8.3%	12.3%	31.2%	34.2%	2%	19.4%
	Posterior	5.2%	20.4%	20.3%	2.6%	1%	24.9%
	Panuveitis	41.7%	26.5%	17.2%	30.2%	6%	7.8%
	Bilaterality	59.3%	63%	48.4%	86.6%	79%	67.3%
Commonest causes and complications	JIAU	35.5%	12.2%	6.2%	25%	56%	19.9%
	Idiopathic	46.9%	51%	50%	50%	24.5%	12.8%
	Behçet’s disease	11.4%	6.1%	6.2%	19.7%	2.7%	2.9%
	Most common complication (%)	Posterior synechiae (12.5%)	Posterior synechia (32.5%)	Optic disc edema (32.6%)	Glaucoma (7.7%)	Posterior synechiae (27%), vision loss (27%)	NA

The etiology of pediatric uveitis is generally subdivided into infectious causes, noninfectious causes, and masquerade syndromes. The most common infectious causes worldwide are toxoplasmosis, toxocariasis, viral infections, and tuberculosis (in developing countries), while noninfectious causes predominate the etiology of uveitis. The most frequent causes are systemic autoimmune diseases such as JIA, BD, sarcoidosis, tubulointerstitial nephritis, and uveitis (TINU) syndrome, and Vogt-Koyanagi-Harada syndrome (VKH). If the cause of uveitis cannot be identified, it is classified as idiopathic [19,20].

At our joint clinic, every uveitis patient undergoes an etiological workup, including a thorough history, physical examination, detailed slit-lamp examination (Figure 1), and laboratory workup to identify the cause of noninfectious uveitis. In our cohort, the most frequent identifiable causes were JIA and BD in 46.9% and 11.4%, respectively. Throughout the study period, 46.9% of the patients were labeled as idiopathic, as shown in Table 1. One explanation of the more common uveitis in JIA may be related to regular uveitis screening in JIA patients and the retrospective nature of this study.

The common noninfectious uveitis etiologies are widely variable between reports from different countries, but idiopathic causes are the most common in most reports [13,15,16,18]. Only Morelle et al. reported idiopathic causes in 24.5% of cases [17]. This etiological variation of non-idiopathic causes might be explained by the ethnic and geographic distribution of reports from populations worldwide in Europe, Asia, and North America [21].

Complications may occur during the disease course and may be due to the disease itself or treatment modalities. We observed complications in 31% of patients listed in Table 3. Posterior synechiae was the most common complication (12.5%), followed by cataract and retinal detachment (6.2% for each one). We compare our findings to regional and international data in Table 4. Posterior synechiae are the most common complication, according to Al-Haddad et al. [15] and Morelle et al. [17]. Still, the rates were much higher than what we have reported by 32.5% and 27%, respectively.

On the other hand, reports from India show that 40%-60% of patients develop complications either at the time of diagnosis or during follow-up, and cataract was the most frequent one [22,23]. Ganesh et al. [23] explained that the higher rates of cataracts are caused by slower referral in India compared to other parts of the world, excessive use of topical corticosteroids, and delay in the use of corticosteroid-sparing therapy. Legal blindness in our patients was reported in only 3.1% and was also less common than that found in previous reports (5.3%-18%) [13,15-18]. The lower rates of complications and the less frequent cataracts in our cohort might be attributed to the early use of DMARDs and the judicious use of topical corticosteroids.

A multidisciplinary approach involving an expert ophthalmologist and pediatric rheumatologist is the optimal treatment approach for noninfectious uveitis in children. However, there is a lack of randomized controlled studies on the treatment of pediatric uveitis, so treatment based on consensus expert opinion or clinical experience and management remains non-standardized. Nevertheless, all guidelines agree on a step-by-step approach where topical corticosteroids are used initially. Still, systemic corticosteroids are used in refractory cases in the presence of uveitis beyond the anterior location. Unresponsive disease necessitates intensifying immunosuppressive treatment with DMARDs with or without biological agents based on the severity and refractoriness of the uveitis [24,25].

Table 2 shows the treatment options used in our patients. Nearly all patients received topical treatment that included mydriatics and topical steroids. Systemic steroids were used in most patients at one point during the treatment strategy, and 15% needed steroid pulse therapy for vision-threatening diseases. Two-thirds used MTX as a DMARD, and one-third used a biological agent due to disease severity or refractoriness to conventional DMARD. Sood and Angeles-Han [24] reported treatment options for pediatric uveitis patients enrolled in the Childhood Arthritis and Rheumatology Research Alliance (CARRAnet) Registry, a large registry of North American pediatric rheumatology patients. They examined practice patterns of pediatric rheumatologists and found that 90% of patients received topical steroids. In comparison, 94% of children were treated with systemic steroids either orally or intravenously during the disease course, but topical and systemic steroids were not used as a long-term treatment.

DMARD options for long-term immunosuppression include first-line treatment with MTX in 76% of patients. In a systemic review and meta-analysis report, Simonini et al. [26] concluded that MTX effectively controls inflammation in pediatric noninfectious uveitis. The proportion of responding subjects in nine eligible studies was 0.73 (95% confidence interval: 0.66-0.1). The less commonly used DMARDs in the CARRAnet Registry were mycophenolate mofetil (MMF) and cyclosporine A (CsA).

In moderate to severe noninfectious uveitis, there are still patients who are refractory to steroids and DMARDs. Biological agents such as tumor necrosis factor inhibitors (TNFi) have emerged as a treatment option. However, adalimumab is the only non-corticosteroid medication approved by the US Food and Drug Administration (FDA) to treat noninfectious uveitis. However, infliximab has been commonly used with excellent success.

A recent review by Norcia et al. [27] showed that adalimumab was superior to placebo in reducing inflammatory activity and steroid use. There was no difference between adalimumab and infliximab in response to treatment. However, the APTITUDE trial by Ramanan et al. [28] showed that 30%-40% of patients with JIA-associated uveitis were refractory to both MTX and TNFi. The data were encouraging for using the anti-IL6 monoclonal antibody tocilizumab in such cases. Fortunately, in our cohort, patients who were refractory to DMARD and infliximab were responsive to adalimumab.

One of the limitations of our study is its retrospective nature. Furthermore, it was conducted in a single center rather than being population-based, which would have given more details about pediatric noninfectious uveitis. Lastly, all patients were evaluated and followed up at a tertiary referral center, which might have affected the etiological classification as the non-referred mild to moderate cases were not included.

## Conclusions

In conclusion, to the best of our knowledge, this is the first study in Jordan describing the clinical spectrum of pediatric noninfectious uveitis. Our study shows that bilateral anterior uveitis was reported in the majority of noninfectious uveitis cases, and idiopathic uveitis was the most common classification. Early commencement of systemic conventional immunosuppressive treatment and prompt use of biological treatment in severe cases improved our cohort’s outcomes and decreased complications. We believe that noninfectious uveitis in Jordanian children is still underdiagnosed or overlooked, and improving awareness among healthcare professionals who work with pediatric populations might improve early recognition and referral.
